# Effects of Statin Therapy on Clinical Outcomes of Survivors of Acute Myocardial Infarction with Severe Systolic Heart Failure

**DOI:** 10.1371/journal.pone.0144602

**Published:** 2015-12-11

**Authors:** Jong Shin Woo, Seung Joon Hwang, Hyun Soo Kim, Jin Bae Kim, Woo-Shik Kim, Kwon Sam Kim, Myung Ho Jeong, Weon Kim, Korea Acute Myocardial Infarction Registry Investigators

**Affiliations:** 1 Division of Cardiology, Department of Internal Medicine, Kyung Hee University Hospital, Kyung Hee University, Seoul, Korea; 2 Division of Cardiology, Department of Medicine, Chonnam National University Hospital, Gwangju, Korea; University of Manitoba, CANADA

## Abstract

**Objective:**

Large randomized trials have failed to show a beneficial effect of statin treatment in chronic HF. The investigators tried to evaluate the long-term effects of statin therapy in patients with new onset heart failure (HF) following acute myocardial infarction (AMI).

**Methods:**

Between January 2008 and December 2011, a total of 13,616 AMI patients were enrolled in the Korea Acute Myocardial Infarction Registry (KAMIR) which was a prospective, multi-center, nationwide, web-based database of AMI in Korea. From this database, we studied 1,055 patients with AMI who had newly developed severe acute HF [left ventricular ejection fraction ≤ 40%] and were discharged alive. The patients were divided into two groups, a statin group (n = 756) and a no-statin group (n = 299). We investigated the one-year major adverse cardiovascular events (MACEs), including all-cause mortality, MI, and any revascularization of each group. We then performed a propensity-score matched analysis.

**Results:**

In the original cohort, one-year MACEs were similar between the two groups (16.5% vs. 14.7% in the statin or no-statin groups; *p* = 0.47). Propensity-score matching yielded 256 pairs, and in that population we observed comparable results in terms of MACEs (18.0% vs. 12.5% in the statin or no-statin groups, *p* = 0.11) and mortality (5.1% vs. 3.5% in the statin or no-statin groups, *p* = 0.51). Cox-regression analysis revealed that statin therapy was not an independent predictor for occurrence of a MACE [Hazard ratio (HR) 1.11, 95% CI 0.79–1.57, *p* = 0.54] or all-cause mortality (HR 1.42, 95% CI 0.75–2.70, *p* = 0.28).

**Conclusion:**

Statin therapy was not associated with a reduction in the long-term occurrence of MACEs or mortality in survivors of AMI with severe acute HF in this retrospective cohort study.

## Introduction

The 3-hydroxy-3-methylglutaryl coenzyme-A inhibitors or statins are widely used, and statin therapy has been shown to reduce adverse cardiovascular (CV) events and all-cause mortality in atherosclerotic coronary artery disease [1,2]. However, most clinical trials of statins have excluded patients with acute or chronic heart failure (HF), so the effects of statin therapy on outcomes in HF remain unclear. Although some clinical studies have shown that statin therapy is associated with improved outcomes in patients with chronic HF and that statin therapy reduces the development of HF after acute coronary syndrome [3,4,5,6], two large randomized clinical trials failed to demonstrate a beneficial effect of statin add-on therapy (rosuvastatin) in chronic HF patients who were medically well-controlled [7,8]. Beyond the lipid-lowering effect of statins, there are abundant pleiotropic effects such as improving endothelial function, enhancing stabilization of atheromatous plaque, decreasing oxidative stress, and decreasing vascular inflammation which would be translated to immediate benefits for patients with acute myocardial infarction (AMI) [9,10]. Even statin therapy would not be beneficial in patients with chronic and established HF, we hypothesized that there would be different results in new onset HF patients by its pleiotropic effects. Additionally, there is a lack of data demonstrating the effect of on clinical outcomes of statin therapy on clinical outcomes in patients with severe acute HF who survived an AMI. To address this issues, we performed an observational study using a nationwide registry to ascertain whether statin therapy improves long-term clinical outcomes, including major adverse cardiac events (MACEs) and mortality, in survivors from acute ischemic HF. We also analyzed clinical outcomes of acute ischemic HF patients by intensity of statin dosage.

## Methods

### Ethics statement

Our study was conducted according to the ethical guidelines of the 1975 Declaration of Helsinki, as revised in 2000. The study protocol was approved by the institutional review board of all centers, and the approval number was 05–49 of Chonnam National University Hospital. Participating sites are listed in an appendix. Given the retrospective design of the project, this institutional review board waived the need for consent. The study was conducted using the format recommended by the Strengthening the Reporting of Observational Studies in Epidemiology (STROBE) guidelines [11].

### Data Source

**Fig 1:**
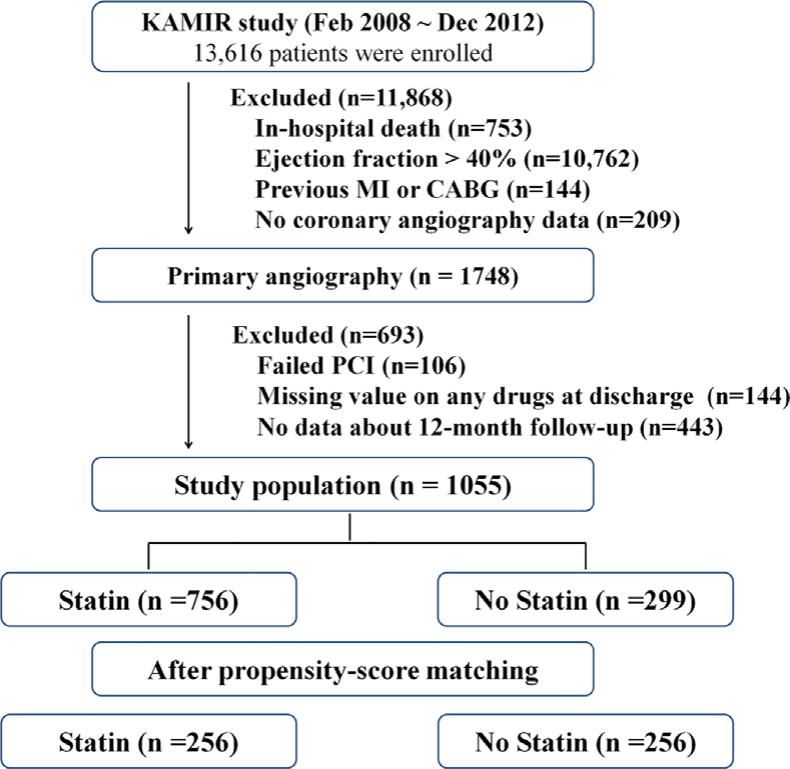
Study flow chart. KAMIR, The Korea Acute Myocardial Infarction Registry; MI, myocardial infarction; CABG, coronary artery bypass grafting; PCI, percutaneous coronary intervention

The Korea Acute Myocardial Infarction Registry (KAMIR) was developed to establish a standardized practice guideline for AMI patients in Korea. The KAMIR was a prospective, multi-center nationwide web-based database supported by the Korean Society of Cardiology that collects precise data with regard to demographic characteristics, treatment strategies in the emergency room, detailed angiographic findings, in-hospital medications, and the clinical outcomes of AMI patients. The detailed was described in previously [12]. It began in January 2008, and 53 centers that were capable of performing a large number of primary percutaneous coronary interventions (PCIs) participated. As shown in Fig 1, a total of 443 (25%) patients were excluded this study because of omitting 12-month clinical follow-up data. In this study, the data completeness of demographics including previous medical history were 97% and 85~95% for laboratory data. But discharge medications, clinical follow-up data were completed.

### Study Design and Population

This is a retrospective observational pooled analysis of nationwide registry data. Between January 2008 and December 2011, a total of 13,616 AMI patients were enrolled in the KAMIR registry. As shown in Fig 1, a total of 1,748 patients with a left ventricular ejection fraction (LVEF) less than 40% were eligible. Patients who had a past medical history of previous myocardial infarction (MI) or coronary artery bypass grafting (CABG) surgery were not included in order to exclude patients with chronic HF. Additionally, we did not include patients who died in hospital in order to only include AMI survivors in the study. We also excluded patients with missing data about medication and clinical follow-up. Ultimately, 1,055 patients with newly developed severe ischemic HF who presented with AMI were enrolled in this study. The patients were then divided into the following two groups according to the whether a statin was included among their discharge medications: treated with a statin group (statin group, n = 756) and not treated with a statin group (no-statin group, n = 299). The mean study follow-up duration was 336 ± 78 days. We also performed a propensity score matched analysis for treatment with statin as described in below.

### Study outcomes

All of the definitions of the clinical outcomes in this study were based on the recommendations of Academic Research Consortium [13]. The primary endpoint of this study was a 12-month composite of MACEs, which was composed of all-cause mortality, recurrent MI, and any repeat PCI or coronary artery bypass grafting (CABG). All-cause mortality included cardiac and noncardiac deaths. Recurrent MI was defined as recurrent symptoms with new electrocardiographic changes compatible with MI or cardiac markers at least twice the upper limit of normal. Stent thrombosis was defined as definite and probable stent thrombosis according to the Academic Research Consortium definition [13]. Target lesion revascularization was defined as any repeat PCI of the target lesion or coronary bypass surgery of the target vessel or other complications related to the target lesion. The target lesion was defined as the treated segment within a stent or within 5 mm proximal or distal to the stent. Target vessel revascularization was defined as any repeat PCI or coronary bypass surgery of any segment of the target vessel. The target vessel was defined as the entire major coronary vessel proximal and distal to the target lesion, which included upstream and downstream branches and the target lesion itself. The secondary endpoint included clinical outcomes according to the intensities of statin therapy.

### Statistical Analysis

All descriptive data are expressed as the mean ± SEM. To compare continuous and categorical variables between groups, Student’s *t*-test (2-sided) and Chi-square test or Fisher's exact test were used, respectively. An unadjusted Cox proportional-hazards model was used to evaluate the association between statin treatment and clinical outcomes and to calculate hazard ratios (HR) with 95% confidence intervals (CI). The cumulative MACE and mortality rates were estimated using the Kaplan-Meier method with the log rank test, and the time-dependent effect of statin therapy as a covariate on clinical outcomes was analyzed using a Cox proportional hazards regression model.

To overcome differences in baseline characteristics and discharge medications between the two groups and to minimize any selection bias, we performed a propensity-score matched analysis, which yielded 256 well-matched pairs. Briefly, the propensity-score of each patient, which indicated the estimated probability of receiving statin treatment, was calculated from the equation of a logistic regression model. Variables included in the logistic regression model used to deduce the propensity-score were age; gender; body mass index; systolic and diastolic blood pressure; heart rate; hypertension; diabetes mellitus; dyslipidemia; family history of heart disease; smoking history; specific medical history, including coronary artery disease, cerebrovascular accident, peripheral artery disease, and statin use before index admission; and lipid profiles, including total cholesterol, triglyceride, low-density lipoprotein (LDL) cholesterol, and high-density lipoprotein cholesterol. The c-statistic for the propensity score derivation was 0.85. Analyses were carried out using SPSS for Windows, version 19.0 (SPSS Inc., Chicago, Illinois).

## Results

### Baseline characteristics, angiographic results and medications

Baseline demographic characteristics and laboratory findings of the patients are demonstrated in Table 1. Systolic blood pressure (BP), diastolic BP, medical history of hyperlipidemia, and previous statin use were significantly different between the two groups. Among laboratory findings, the serum creatinine level was significantly lower in the statin group. However, serum total cholesterol, triglyceride and LDL cholesterol levels were significantly higher in the statin group. LVEF measured by echocardiography was not different between the two groups (33.8 ± 5.9% in the statin group vs. 33.9 ± 6.2% in the no-statin group, *p* = 0.72). In Angiographic results, lesion characteristics showed more severe in the statin group, but pre-procedural Thrombolysis in Myocardial Infarction score; pre-procedural lesion stenosis; and implanted stent number, diameter, and length were not significantly different between the two groups (S1 Table). In the propensity score-matched populations, all baseline characteristics, laboratory findings, and angiographic results were balanced between the two groups. In discharge medications, statin group patients more frequently had aspirin and clopidogrel among their discharge medications. However, in the propensity score-matched populations, discharge medications were not significantly different between the two groups.

**Table 1:** Baseline characteristics.

	Total population			Propensity-matched population		
	No Statin(n = 299)	Statin(n = 756)	*p* value	No Statin(n = 256)	Statin(n = 256)	*p* value
Age	66.6 ± 11.7	66.8 ± 12.4	0.83	66.7 ± 11.5	66.2 ± 12.0	0.64
Gender (male)	218 (73%)	517 (68%)	0.15	72 (28%)	72 (28%)	0.99
Body mass index (kg/m^2^)	23.3 ± 3.5	23.5 ± 3.2	0.35	23.1 ± 3.5	22.9 ± 2.8	0.54
Systolic blood pressure (mmHg)	122.6 ± 31.6	127.8 ± 26.9	< 0.01	124.0 ± 28.7	127.8 ± 27.1	0.13
Diastolic blood pressure (mmHg)	76.2 ± 19.9	78.6 ± 16.6	0.04	77.1 ± 18.2	78.1 ± 15.7	0.53
Heart rate (bpm)	84.1 ± 23.3	86.5 ± 21.4	0.11	84.4 ± 22.5	85.9 ± 21.4	0.45
Final diagnosis (STEMI)	172 (57%)	452 (60%)	0.50	103 (40%)	110 (43%)	0.59
Killip class (more than II)	140 (49%)	316 (44%)	0.15	124 (50%)	109 (44%)	0.18
Previous statin use	14 (5%)	62 (25%)	0.03	11 (4%)	9 (3%)	0.82
**Cardiovascular risk factors**						
Hypertension	158 (53%)	410 (55%)	0.61	136 (53%)	133 (52%)	0.86
Diabetes mellitus	110 (37%)	255 (34%)	0.36	94 (37%)	97 (38%)	0.85
Hyperlipidemia	14 (5%)	112 (15%)	< 0.01	10 (4%)	12 (5%)	0.83
Current smoking	122 (42%)	284 (38%)	0.29	108 (42%)	108 (42%)	0.99
Previous coronary artery disease	46 (15%)	144 (19%)	0.16	38 (15%)	34 (13%)	0.70
**Laboratory findings**						
Ejection fraction	33.9 ± 6.2	33.8 ± 5.9	0.72	34.3 ± 6.0	34.3 ± 5.8	0.89
Glucose (mg/dL)	194.9 ± 103.4	187.7 ± 93.9	0.27	201.1 ± 110.4	197.1 ± 105.5	0.68
Creatinine (mg/dL)	1.4 ± 1.7	1.2 ± 1.1	0.02	1.2 ± 1.2	1.3 ± 1.6	0.38
Max-CK-MB (mg/dL)	154.6 ± 213.6	144.3 ± 184.7	0.44	146.8 ± 189.4	157.4 ± 213.1	0.55
Max-cTnI (mg/dl)	79.5 ± 250.4	72.2 ± 190.1	0.64	83.0 ± 181.2	77.8 ± 234.3	0.79
Total cholesterol (mg/dL)	169.1 ± 36.9	182.9 ± 48.4	< 0.01	170.9 ± 45.6	168.8 ± 36.4	0.58
Triglyceride (mg/dL)	105.6 ± 63.3	120.3 ± 86.0	< 0.01	109.1 ± 68.9	103.5 ± 62.5	0.34
HDL-cholesterol (mg/dL)	45.5 ± 26.7	44.1 ± 12.9	0.28	44.0 ± 14.0	45.6 ± 27.8	0.43
LDL-cholesterol (mg/dL)	108.4 ± 58.1	117.9 ± 41.9	< 0.01	105.4 ± 38.3	104.6 ± 33.8	0.80
hsCRP (mg/dL)	7.7 ± 25.1	8.1 ± 26.6	0.81	11.3 ± 36.8	8.3 ± 26.6	0.31
NT-proBNP (ng/dL)	5510.2 ± 8927.4	4915.7 ± 7700.0	0.35	5853.1 ± 9139.0	5693.0 ± 9213.9	0.86
HbA1C (%)	6.8 ± 1.7	6.6 ± 1.5	0.13	6.7 ± 1.4	6.8 ± 1.7	0.37
**Discharge medications**						
Aspirin	289 (97%)	753 (99%)	< 0.01	250 (98%)	254 (99%)	0.28
Clopidogrel	287 (96%)	745 (98%)	0.01	248 (97%)	251 (98%)	0.58
Calcium-channel blocker	14 (5%)	42 (6%)	0.57	13 (5%)	18 (7%)	0.46
Beta-blocker	237 (79%)	627 (83%)	0.16	208 (81%)	208 (81%)	0.99
ACE inhibitor	158 (53%)	435 (57%)	0.17	136 (53%)	144 (56%)	0.53
Angiotensin-receptor blocker	80 (27%)	199 (26%)	0.88	71 (28%)	62 (24%)	0.42
**Angiographic findings**						
Stenotic vessels						
1 vessel disease	93 (32%)	258 (35%)	0.38	81 (32%)	85 (33%)	0.78
2 vessel disease	88 (30%)	234 (31%)	0.67	75 (29%)	80 (31%)	0.70
3 vessel disease	97 (33%)	233 (30%)	0.32	87 (34%)	80 (21%)	0.57
Left main coronary artery involved	15 (5%)	30 (4%)	0.44	13 (5%)	11 (4%)	0.83
ACC/AHA lesion class						
Type A or B1	98 (32%)	113 (17%)	< 0.01	83 (32%)	80 (31%)	0.55
Type B2 or C	201 (67%)	554 (83%)	< 0.01	173 (68%)	176 (69%)	0.55
Pre TIMI flow 0/1	183 (66%)	425 (60%)	0.38	148 (58%)	136 (56%)	0.65
Post TIMI flow 3	246 (93%)	647 (94%)	0.59	218 (93%)	226 (95%)	0.44
Target stent length	24.2 ± 6.1	24.4 ± 6.9	0.59	24.1 ± 6.6	24.1 ± 6.1	0.85
Target stent diameter	3.1 ± 0.4	3.1 ± 0.4	0.89	3.1 ± 0.4	3.1 ± 0.4	0.77
Total stent number	1.5 ± 0.9	1.6 ± 0.8	0.40	1.6 ± 0.8	1.5 ± 0.9	0.49

CK-MB, creatine kinase MB; cTnI, cardiac troponin I; cTnT, cardiac troponin T; GFR, glomerular filtration rate; HDL, high-density lipoprotein; hsCRP, high-sensitivity C-reaction protein; LDL, low-density lipoprotein; NT-pro BNP, N-terminal prohormone of brain natriuretic peptide; ACC/AHA, American College of Cardiology/American Heart Association; TIMI, Thrombolysis in Myocardial Infarction

### The effects of statin therapy on clinical outcomes

**Fig 2:**
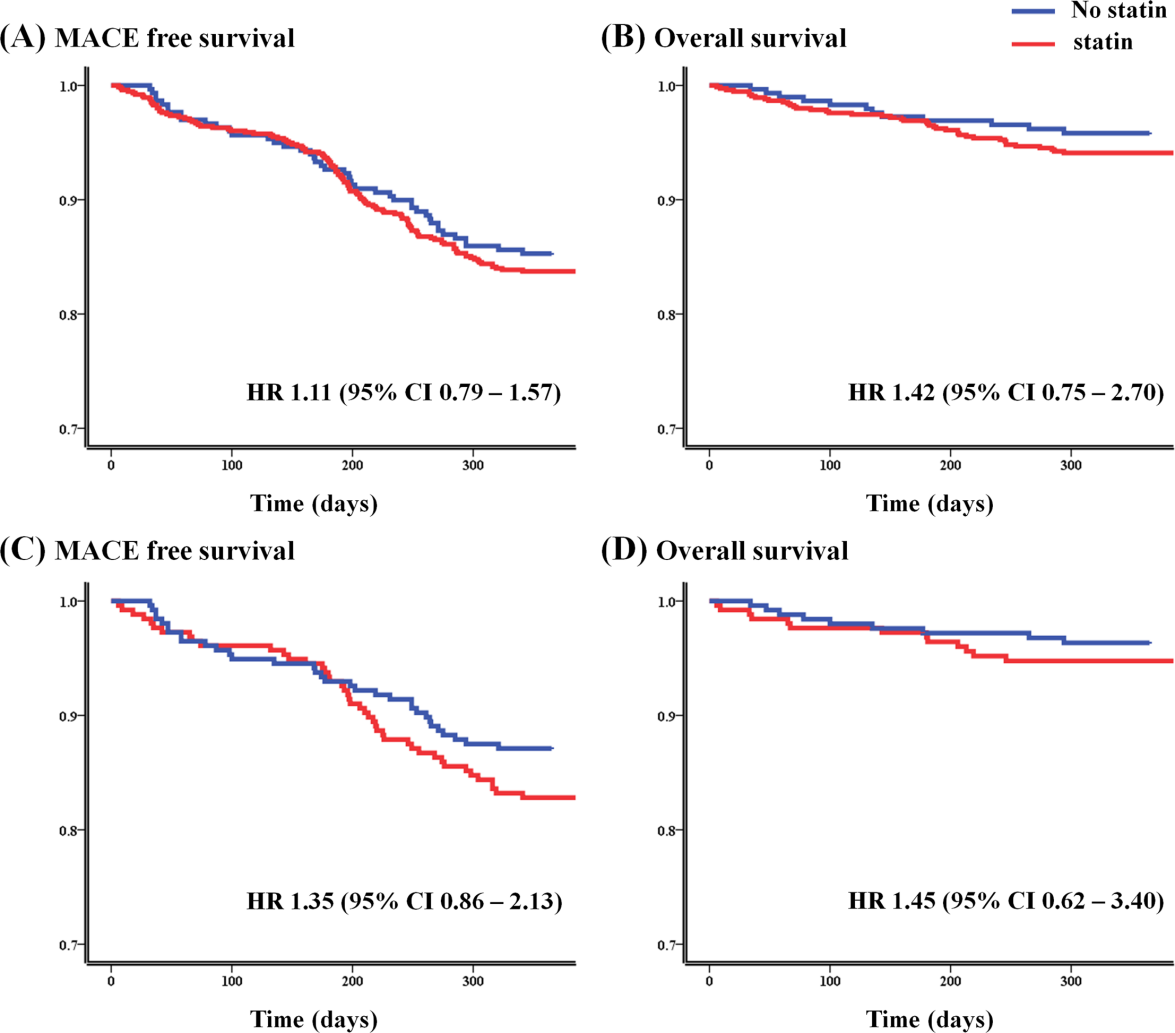
Event-free survival curves. The Kaplan-Meier curves demonstrate no significant differences between statin and no-statin groups for either 12-month major adverse cardiovascular events (A, overall population; C, propensity-score matched population) or 12-month all cause mortalities (B, overall population; D, propensity-score matched population). MACE, major adverse cardiovascular event; HR, hazard ratio

As shown in Table 2, the prevalence of one-year MACEs and one-year all-cause mortality were not significantly different between the two groups (Fig 2A and 2B). Similarly, one-year MACE-free and one-year all-cause survival rates in the propensity score-matched populations were not statistically different (Fig 2C and 2D). Cox proportional hazard regression analysis revealed that long-term statin use, as indicated by its inclusion in the discharge medications, did not reduce the incidence of MACEs or all-cause mortality during follow-up. Likewise, in the propensity-score matched populations, statin use did not reduce the incidence of MACEs or all-cause mortality during one-year of follow-up.

**Table 2:** Overall clinical outcomes.

	Total population			Propensity-matched population		
	No Statin (n = 299)	Statin (n = 756)	*p* value	No Statin (n = 256)	Statin (n = 256)	*p* value
Overall MACE^*^	44 (14.7%)	125 (16.5%)	0.47	32 (12.5%)	46 (18.0%)	0.11
All-cause mortality	12 (4.0%)	43 (5.7%)	0.27	9 (3.5%)	13 (5.1%)	0.51
Cardiac death	9 (3.0%)	32 (4.2%)	0.35	6 (2.3%)	9 (3.5%)	0.60
Non-cardiac death	3 (1.0%)	11 (1.5%)	0.56	3 (1.2%)	4 (1.6%)	0.99
Myocardial infarction	6 (2.0%)	16 (2.1%)	0.91	6 (2.3%)	5 (2.0%)	0.99
Repeated Revascularization	24 (8.0%)	55 (7.3%)	0.68	17 (6.6%)	23 (9.0%)	0.41
TLR	12 (4.0%)	28 (3.7%)	0.81	10 (3.9%)	13 (5.1%)	0.67
TVR	4 (1.3%)	8 (1.1%)	0.70	4 (1.6%)	2 (0.8%)	0.69
CABG	2 (0.7%)	6 (0.8%)	0.83	1 (0.4%)	0 (0%)	0.99
Stent thrombosis	1 (0.3%)	7 (0.9%)	0.32	1 (0.4%)	5 (2%)	0.22

^*^Composed of all-cause mortality, myocardial infarction and repeated revascularization.

CABG, coronary artery bypass grafting; CI, confidence interval; HR, hazard ratio; MACE, major adverse cardiovascular event; TLR, target-lesion revascularization; TVR, target-vessel revascularization

The statins prescribed at discharge and the intensities of statin therapy are demonstrated in Table 3. Atorvastatin was the most common statin included in the discharge medication lists, followed by rosuvastatin, pitavastatin, and simvastatin. However, the kind of statin did not have a significant effect on clinical outcomes in terms of MACEs or all-cause mortality (*p* = 0.70, 0.88, respectively). The intensities of statin therapy were also not an independent predictor of MACEs or all-cause mortality (*p* = 0.79, 0.43, respectively), and the same tendency was observed in the propensity score-matched populations.

**Table 3:** The effects of statin therapy on clinical outcomes.

	Total population		Propensity-matched population	
	*Composites of MACE	All-cause mortality	*Composites of MACE	All-cause mortality
**Kinds of statin**				
Atorvastatin	74 / 432 (17.1%)	25 / 432 (5.8%)	29 / 145 (20.0%)	6 / 145 (4.1%)
Rosuvastatin	26 / 193 (13.5%)	12 / 193 (6.2%)	9 / 63 (14.3%)	5 / 63 (7.9%)
Pitavastatin	12 / 68 (17.6%)	2 / 68 (2.9%)	5 / 28 (17.9%)	0 / 28 (0%)
Simvastatin	9 / 41 (22.0%)	2 / 41 (4.9%)	1 / 13 (7.7%)	0 / 13 (0%)
Pravastatin	3 / 17 (17.6%)	2 / 17 (11.8%)	2 / 7 (28.6%)	2 / 7 (28.6%)
Fluvastatin	1 / 5 (20.0%)	0 / 5 (0%)	-	-
**Intensities of statin**				
Low	2 / 11 (18.2%)	1 / 11 (9.1%)	1/6 (16.7%)	1/3 (33.3%)
Moderate	102 / 615 (16.6%)	33 / 615 (5.4%)	21/222 (9.5%)	8/213 (3.8%)
High	21 / 130 (16.2%)	9 / 130 (6.9%)	5/62 (8.1%)	4/40 (10.0%)

*Composed of all-cause mortality, myocardial infarction (MI), any repeated revascularization. MACE, major adverse cardiovascular event

## Discussion

In this registry-based observational study, we found that long-term statin therapy did not reduce one-year MACEs or all-cause mortality in patients with AMI complicated by severe acute HF. Additionally, neither the type nor the intensity of statin therapy affected the one-year clinical outcomes of enrolled patients.

Although all of the patients in our study suffered from new onset acute HF, the results of our study are comparable to other prospective randomized controlled trials, such as Controlled Rosuvastatin Multinational Trial in HF trial (CORONA) or GISSI-Heart Failure trial (GISSI-HF), that enrolled patients with chronic systolic HF [7,8]. In contrast, several studies suggested that statin therapy could reduce MACEs in patients with acute ischemic HF complicating AMI. The post-hoc analysis of Eplerenone Post-Acute Myocardial Infarction Heart Failure Efficacy and Survival (EPHESUS) study revealed that all-cause mortality occurred more in patients belonging to the group that did not receive a statin than the group that received a statin (18% vs. 12%, *p* < 0.01), and after adjustment for various confounding factors, the relative risk of all-cause mortality was lower in patients belonging to the group that received statin therapy (HR 0.80, 95% CI 0.69–0.92, *p* < 0.01) [14]. In addition, a small retrospective study demonstrated a lower all-cause mortality rate in the group that was prescribed a statin at discharge compared with a no-statin group (28.5% vs. 53.3%, *p* < 0.01). Statin therapy has also been shown to independently reduce HF re-admissions (7.0% vs. 32.0%, *p* < 0.01) and death from HF (4.0% vs 13.0%, *p* < 0.01) [15]. However, these reports are retrospective and have small sample sizes, and there is a lack of randomized data demonstrating the efficacy of statin therapy in patients with AMI complicated by acute HF.

Recent clinical studies have suggested that the potential benefits of statins in HF patients can be partially explained by their lipid-independent pleiotropic effects. These pleiotropic effects include stabilizing atherosclerotic plaques, improving endothelial function, and alleviating vascular oxidation and inflammation [16]. On the other hand, the mechanisms underlying the harmful effects of statin therapy in HF patients remain poorly understood. Some studies have demonstrated that low serum cholesterol levels in HF patients are related to adverse clinical outcomes [17,18], but whether a low serum cholesterol level itself induces deterioration of HF is unclear. Additionally, it has been proposed that statin therapy could increase susceptibility to infections in patients with HF by diminishing lipoproteins that eliminate bacterial lipopolysaccharides [19]. Other researchers have suggested that statins could damage cardiac myocytes by lowering levels of serum coenzyme Q_10_. Coenzyme Q_10_ is a key element in mitochondrial oxidative phosphorylation, and its depletion has been reported to be associated with increased mortality [20,21], but McMurray *et al*. demonstrated that neither statin (rosuvastatin) nor coenzyme Q_10_ is an independent prognostic factor in patients with HF [22].

According to the latest guidelines for dyslipidemia management, there is no obvious evidence that supports a beneficial effect of statin therapy in patients with New York Heart Association (NYHA) class II-IV HF [23,24]. Additionally, in the International Atherosclerosis Society guideline and National Institute for Health and Care Excellence guideline, there is no special mention of statin use in patients with HF [25,26]. According to this observational study and previous two large randomized trials [7,8], there was no benefit from initiating statin therapy in patients with severe HF (mean LVEF were less than 33 percent). But this results could not be generalized to mild HF patients or HF with preserved EF. Further prospective randomized controlled trials are needed to validate the role of statin therapy in acute ischemic HF patients.

Our study had a number of limitations. First, it was a retrospective, observational study based on a registry data. There was a lack of data that cannot be ignored although we encouraged to fill-up the missing values. Undetected selection bias may remain despite our cautious use of propensity-score matching with excluding patients with missing data. We could not controlled non-cardiac related medications and other patients' condition such as nutritional status or life style because of inherent limitations of registry data. Second, the number of patients participating in this study was relatively small. Third, there was the possibility of including patients with pre-existing non-ischemic HF despite of our careful review. Fourth, we have no detailed follow-up data with regard to lipid profiles or echocardiographic parameters of registered patients. Finally, we were not able to acquire safety information for the statins prescribed to the patients, such as the incidence rates of serious adverse effects or compliance data. Despite these drawbacks, we are able to present some negative evidence with regard to statin therapy in patients with MI complicated by severe acute ischemic HF. We also found that the type of statin and intensity of statin therapy did not exert an influence on clinical outcomes.

In conclusion, statin therapy had no significant effect on the one-year incidence of MACEs or mortality in survivors with acute severe ischemic HF following AMI.

## Appendix

### Institutional Review Board of Participating Sites

The Korea Acute Myocardial Infarction Registry (KAMIR) was approved by the institutional review board (IRB) at each participating center (Kyunghee University IRB, Asan Medical Center IRB, Ajou University Hospital IRB, Busan Hanseo Hospital IRB, Busan National University Hospital IRB, Choongbook National University Hospital IRB, Chungnam National University Hospital IRB, Catholic University Hospital IRB, Chonbuk National University Hospital IRB, Chosun University Hospital IRB, Chung-Ang University Hospital IRB, Dankook University Hospital IRB, Daegu Catholic University Medical Center IRB, Dong-A University Medical Center IRB, Daejeon St. Mary's Hospital IRB, Dongguk University Gyeongju Hospital IRB, Gyeongsang National University Hospital IRB, Hallym University Kandong Sacred Heart Hospital IRB, Hallym University Sacred Heart Hospital IRB, Inje University Haeundae Paik hospital IRB, Inje University Sanggye Paik Hospital IRB, Inha University Hospital IRB, Jeju National University Hospital IRB, Kyungpook National University Hospital IRB, Keimyung University Hospital IRB, Korea University Guro Hospital IRB, Konyang University Hospital IRB, Kwangju Christian Hospital IRB, Maryknoll Medical Center IRB, National Health Insurance Corporation Ilsan Hospital IRB, Yeungnam University Hospital IRB, Yonsei University Severance Hospital IRB, Yonsei University Wonju Hospital IRB, Presbyterian Medical Center IRB, Seoul National University Hospital IRB, Seoul National University Bundang Hospital IRB, Soon Chun Hyang University Bucheon Hospital IRB, Soon Chun Hyang University Cheonan Hospital IRB, Samsung Medical Center IRB, Sun General Hospital IRB, Saint Carollo Hospital IRB, Wonkwang University Hospital IRB and Chonnam National University Hospital IRB).

## Supporting Information

S1 Table Angiographic details. (DOC)
