# Supplementary material for: Effects of Statin Therapy on Clinical Outcomes of Survivors of Acute Myocardial Infarction with Severe Systolic Heart Failure
Source: PLoS One. 2015 Dec 11;10(12):e0144602. doi: 10.1371/journal.pone.0144602 (PMC4676648; doi:10.1371/journal.pone.0144602)
Supplement: S1 Table — (DOC) [file pone.0144602.s001.doc]

**Table S1. Angiographic details**

|  | **Total population** | | ***p* value** | **Propensity-matched population** | | ***p* value** |
| --- | --- | --- | --- | --- | --- | --- |
|  | **No Statin**  **(n=299)** | **Statin**  **(n=756)** |  | **No Statin**  **(n=256)** | **Statin**  **(n=256)** |  |
| Stenotic vessels |  |  |  |  |  |  |
| 1 vessel disease | 93 (32%) | 258 (35%) | 0.38 | 81 (32%) | 85 (33%) | 0.78 |
| 2 vessel disease | 88 (30%) | 234 (31%) | 0.67 | 75 (29%) | 80 (31%) | 0.70 |
| 3 vessel disease | 97 (33%) | 233 (30%) | 0.32 | 87 (34%) | 80 (21%) | 0.57 |
| Left main coronary artery involved | 15 (5%) | 30 (4%) | 0.44 | 13 (5%) | 11 (4%) | 0.83 |
| Target lesion |  |  |  |  |  |  |
| Left anterior descending artery | 171 (59%) | 480 (64%) | 0.08 | 143 (56%) | 147 (57%) | 0.72 |
| Left circumflex artery | 40 (13%) | 97 (13%) | 0.99 | 40 (16%) | 37 (14%) | 0.71 |
| Right coronary artery | 72 (25%) | 155 (21%) | 0.18 | 65 (25%) | 63 (25%) | 0.84 |
| Left main coronary artery | 11 (4%) | 13 (2%) | 0.05 | 8 (3%) | 9 (3%) | 0.80 |
| Lesion and stent characteristics |  |  |  |  |  |  |
| Reference vessel diameter (mm) | 3.1 ± 0.5 | 3.1 ± 0.5 | 0.99 | 3.1 ± 0.5 | 3.1 ± 0.5 | 0.83 |
| Pre-procedural stenosis (%) | 89 ± 15 | 87 ± 17 | 0.09 | 89 ± 14 | 89 ± 14 | 0.95 |
| Lesion length (mm) | 23 ± 12 | 26 ± 13 | 0.01 | 23 ± 12 | 25 ± 12 | 0.22 |
| Target Stent diameter (mm) | 3.1 ± 0.4 | 3.1 ± 0.4 | 0.89 | 3.1 ± 0.4 | 3.1 ± 0.4 | 0.77 |
| Target stent length (mm) | 24.2 ± 6.1 | 24.4 ± 6.9 | 0.59 | 24.1 ± 6.6 | 24.1 ± 6.1 | 0.85 |
| Total implanted stents | 1.5 ± 0.9 | 1.6 ± 0.8 | 0.40 | 1.6 ± 0.8 | 1.5 ± 0.9 | 0.49 |
